# Aflatoxin B1 Up-Regulates Insulin Receptor Substrate 2 and Stimulates Hepatoma Cell Migration

**DOI:** 10.1371/journal.pone.0047961

**Published:** 2012-10-24

**Authors:** Yanli Ma, Qingbin Kong, Hui Hua, Ting Luo, Yangfu Jiang

**Affiliations:** 1 State Key Laboratory of Biotherapy, Section of Signal Transduction and Molecular Targeted Therapy, West China Hospital, Sichuan University, Chengdu, China; 2 Cancer Center, West China Hospital, Sichuan University, Chengdu, China; Aix-Marseille University, France

## Abstract

Aflatoxin B1 (AFB1) is a potent carcinogen that can induce hepatocellular carcinoma. AFB1-8,9-exo-epoxide, one of AFB1 metabolites, acts as a mutagen to react with DNA and induce gene mutations, including the tumor suppressor *p53*. In addition, AFB1 reportedly stimulates IGF receptor activation. Aberrant activation of IGF-I receptor (IGF-IR) signaling is tightly associated with various types of human tumors. In the current study, we investigated the effects of AFB1 on key elements in IGF-IR signaling pathway, and the effects of AFB1 on hepatoma cell migration. The results demonstrated that AFB1 induced IGF-IR, Akt, and Erk1/2 phosphorylation in hepatoma cell lines HepG2 and SMMC-7721, and an immortalized human liver cell line Chang liver. AFB1 also down-regulated insulin receptor substrate (IRS) 1 but paradoxically up-regulated IRS2 through preventing proteasomal degradation. Treatment of hepatoma cells and Chang liver cells with IGF-IR inhibitor abrogated AFB1-induced Akt and Erk1/2 phosphorylation. In addition, IRS2 knockdown suppressed AFB1-induced Akt and Erk1/2 phosphorylation. Finally, AFB1 stimulated hepatoma cell migration. IGF-IR inhibitor or IRS2 knockdown suppressed AFB1-induced hepatoma cell migration. These data demonstrate that AFB1 stimulates hepatoma cell migration through IGF-IR/IRS2 axis.

## Introduction

Hepatocellular carcinoma (HCC) is one of common cancers worldwide, especially in Asia and south Africa. Aflatoxin B1 (AFB1), an ubiquitous contaminant of the human foods in developing world, is a known carcinogen that may increase the risk of HCC. Aflatoxin exposure also synergistically increases the risk of liver cancers in people chronically infected with hepatitis virus, another important risk factor in the etiology of HCC [1]. Once taken by liver cells, AFB forms adducts with hepatic DNA, ribosomal RNA, and proteins. Hepatic AFB1-DNA adducts correlate with hepatic cancer risk [2]. Cytochrome P450 enzymes can oxidize AFB1 into several products thereby either activating or detoxicating AFB1. One of these metabolites, AFB1-8,9-exo-epoxide is mutagenic through reacting with DNA [3]. Measurements of AFB1 metabolites AFP1 and AFM1, and AFB1-DNA adducts (AFB1-N7-Guanine) may help to evaluate individual AFB1 exposure [4]. AFB-N7-guanine adduct in urine may serve as a biomarker of the biologically active AFB.

Given the tight association between AFB1 exposure and risk of liver cancer, blockade of AFB1 bioavailability may be a promising strategy to prevent the development of HCC. In randomized, double-blind, placebo-controlled chemoprevention trial, chlorophyllin, which can block AFB1 bioavailability, has been demonstrated to effectively reduce urinary levels of AFB1-N7-guanine adducts [5]. Oltipraz, another chemopreventine agent, can reduce the bioavailability of AFB1 by inhibiting the metabolism of AFB to its carcinogenic form and promoting the detoxication of its metabolites [6]. Interventions with chlorophyllin or oltipraz may represent practical ways to prevent HCC [7].

The precise mechanisms involved in AFB1-induced hepatocarcinogenesis may be complex. As a mutagen, AFB1 may induce mutation of the tumor suppressor gene *p53* at codon 249, which has important role in the development of HCC. In addition, recent study demonstrates that AFB1 may stimulate the expression of insulin-like growth factor-2 (IGF2) and IGF1 receptor (IGF-IR) [8]. *p53* mutant *p53*-mt249 can increase IGF2 transcription, suggesting that *p53* mutation may be a link between AFB1 and IGF2 [9]. Mounting evidences have demonstrated that the IGF axis is involved in human cancer progression [10], [11]. Changes in the IGF axis also affect the molecular pathogenesis of HCC [12]. Both IGF1 and IGF2 are synthesized and secreted by hepatocytes. IGF1 and IGF2 bind to type-1 IGF receptor (IGF-IR) and initiate a cascade of signaling involving the activation of insulin receptor substrate (IRS)−1, −2, ERK, and PI3′ kinase. Activation of IGF-IR signaling leads to increased DNA synthesis and cell migration. Both *p53* mt249 and HBX can up-regulate IGF-IR expression [13].

**Figure 1:**
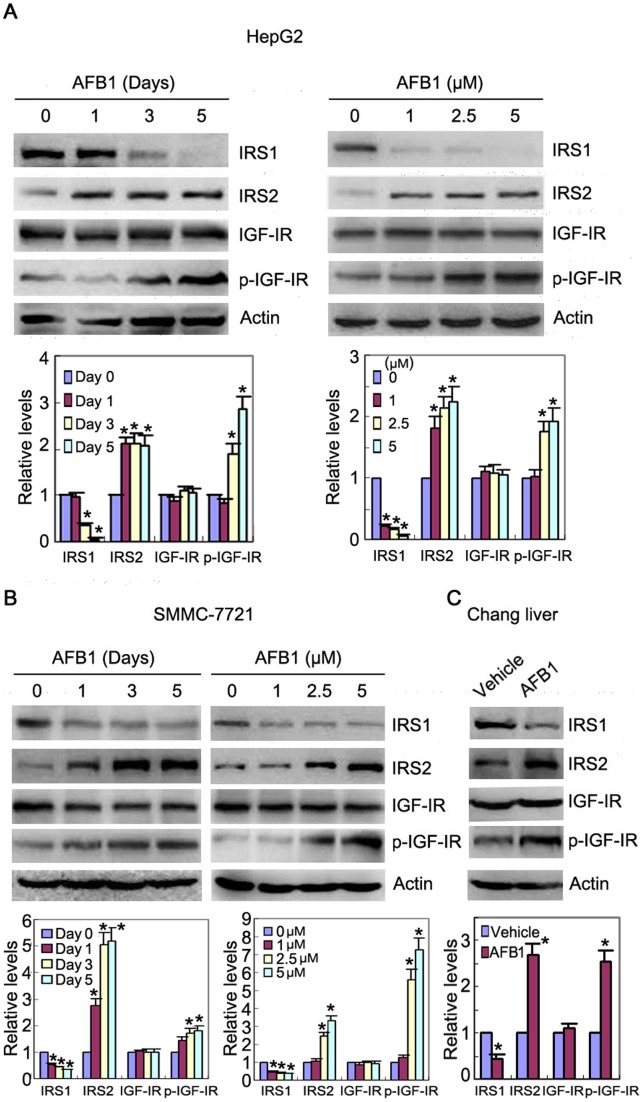
AFB1 induces IGF-IR phosphorylation, down-regulates IRS1 but up-regulates IRS2. (**A**) HepG2 cells were treated with 2.5 µM AFB1 for 1, 3, or 5 days, or treated with 1, 2.5, and 5 µM AFB1 for 3 days, followed by western blot analysis of IGF-IR and phosphorylated IGF-IR, IRS1, and IRS2. (**B**) SMMC-7721 cells were treated with 2.5 µM AFB1 for 1, 3, or 5 days, or treated with 1, 2.5, and 5 µM AFB1 for 3 days, followed by western blot analysis of IGF-IR and phosphorylated IGF-IR, IRS1, and IRS2. (**C**) Chang liver cells were treated with 2.5 µM AFB1 for 3 days, followed by western blot analysis of IGF-IR and phosphorylated IGF-IR, IRS1, and IRS2. All blots were subjected to densitometric analysis. The relative levels of IGF-IR, phosphorylated IGF-IR, IRS1, and IRS2 after normalization to actin were plotted. The relative levels of target proteins in un-treated group were set as 1. A statistical analysis of densitometric quantification of immunoblots from individual experiments was shown. *, *p*<0.05.

To further dissect the effects of AFB1 on IGF-IR signaling, we investigated how AFB1 might regulate the expression and activation of key elements in IGF-IR signaling, including IGF-IR, IRS1, IRS2, ERK and AKT. Here, we report that AFB1 stimulates IGF-IR phosphorylation, down-regulates IRS1, but up-regulates IRS2 expression, which contributes to AFB1-induced hepatoma cell migration.

**Figure 2:**
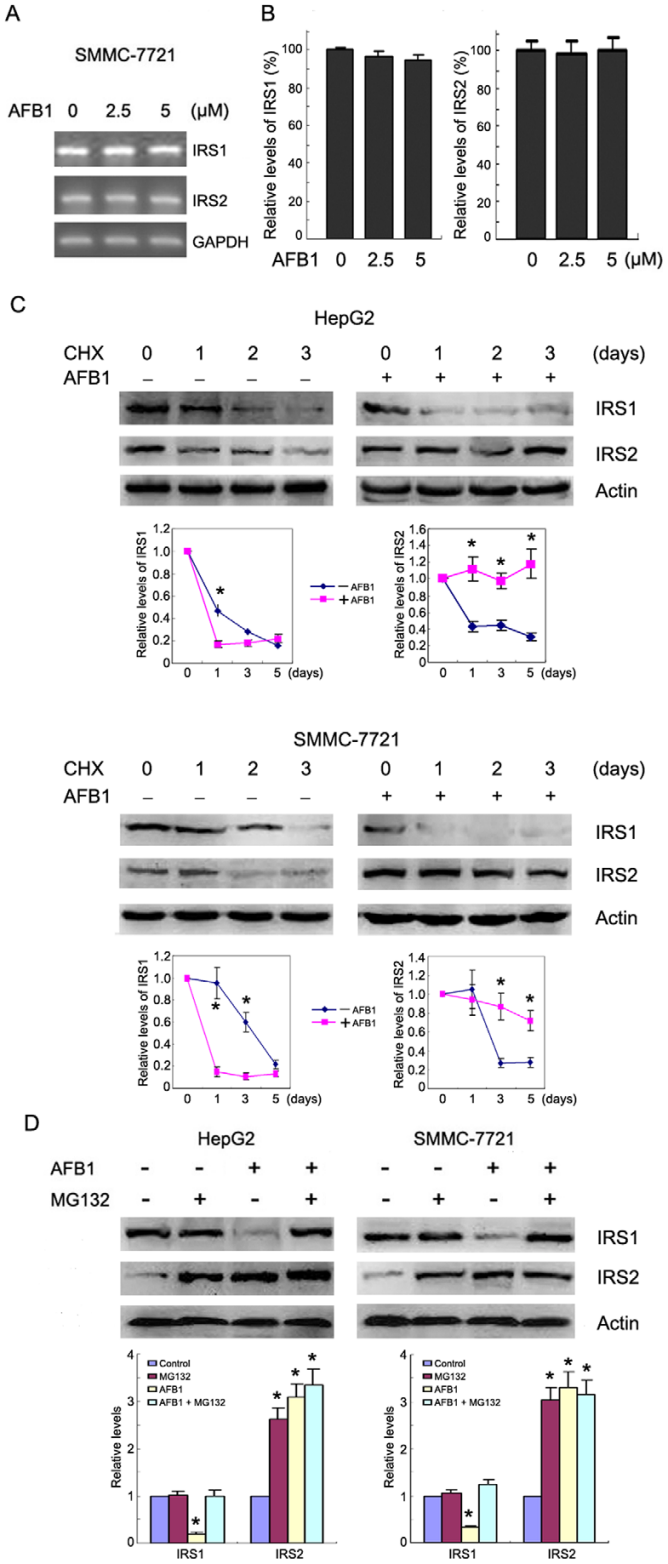
AFB1 does not affect IRS1 and IRS2 transcription but regulates IRS1 and IRS2 degradation. (**A**) SMMC-7721 cells were treated with or without AFB1 at indicated dose for 3 days, followed by RT-PCR analysis of *IRS1* and *IRS2* transcription. *GAPDH* transcription was also detected as internal control. (**B**) SMMC-7721 cells were treated with or without AFB1 at indicated dose for 3 days, followed by real-time RT-PCR analysis of *IRS1* and *IRS2* transcription. The relative *IRS1* or *IRS2* levels were plotted. (**C**) HepG2 and SMMC-7721 cells were treated with 25 µg/ml CHX to inhibit new protein synthesis for the times indicated. In parallel, the cells were treated with combination of AFB1 and CHX. Total proteins were harvested and subjected to western blotting for IRS1, IRS2 and β-actin to control for loading. The blots were subjected to densitometric analysis. The relative levels of IRS1 and IRS2 after normalization to actin were plotted. The relative levels of IRS1 and IRS2 in cells treated without CHX were set as 1. The statistical analysis of densitometric quantification of immunoblots from individual experiments were shown. *, *p*<0.05. (**D**) HepG2 and SMMC-7721 cells were treated with or without 2.5 µM AFB1 and 2 µM proteasome inhibitor MG132 for 3 days, followed by western blot analysis of IRS1 and IRS2 levels. All blots were subjected to densitometric analysis. The relative levels of IRS1 and IRS2 after normalization to actin were plotted. The relative levels of IRS1 and IRS2 in cells treated without AFB1 and MG132 were set as 1. A statistical analysis of densitometric quantification of immunoblots from individual experiments was shown. *, *p*<0.05.

**Figure 3:**
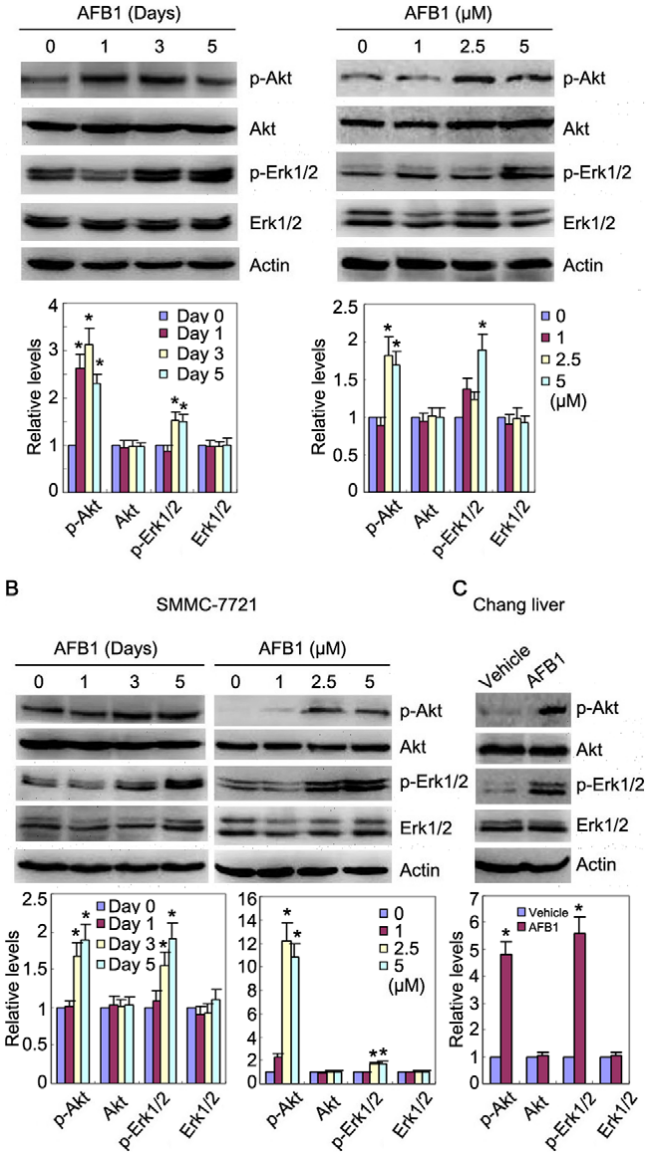
AFB1 induces Akt and Erk1/2 phosphorylation. (**A**) HepG2 cells were treated with 2.5 µM AFB1 for 1, 3, or 5 days, or treated with 1, 2.5, and 5 µM AFB1 for 3 days, followed by western blot analysis of Akt and phosphorylated Akt, Erk1/2 and phosphorylated Erk1/2. (**B**) SMMC-7721 cells were treated with 2.5 µM AFB1 for 1, 3, or 5 days, or treated with 1, 2.5, and 5 µM AFB1 for 3 days, followed by western blot analysis of Akt and phosphorylated Akt, Erk1/2 and phosphorylated Erk1/2. (**C**) Chang liver cells were treated with 2.5 µM AFB1 for 3 days, followed by western blot analysis of Akt and phosphorylated Akt, Erk1/2 and phosphorylated Erk1/2. Immunoblots were subjected to densitometric analysis. The relative levels of Akt and phosphorylated Akt, Erk1/2 and phosphorylated Erk1/2 after normalization to actin were plotted. The relative levels of target proteins in cells treated without AFB1 were set as 1. A statistical analysis of densitometric quantification of immunoblots from individual experiments was shown. *, *p*<0.05.

## Results

### AFB1 induces IGF-IR phosphorylation and IRS2 accumulation

To comprehensively detect the effects of AFB1 on IGF1 signaling pathway, we checked if AFB1 induced any change in the levels of IGF-IR and its substrates, IRS1 and IRS2. Immunoblot analysis demonstrated that treatment of hepatoma cell line HepG2 with AFB1 resulted in an increase in IGF-IR phosphorylation, while the levels of total IGF-IR were unchanged. AFB1 also induced a decrease in the levels of IRS1, but an increase in the levels of IRS2 (Figure 1A). Similar findings were detected in another hepatoma cell line, SMMC-7721 (Figure 1B). In addition, treatment of Chang liver cells, an immortalized human liver cell line, with AFB1 led to an increase in IGF-IR phosphorylation and IRS2 expression but a decrease in IRS1 expression (Figure 1C).

To determine whether AFB1 regulated *IRS1* and *IRS2* at the transcription level, we run RT-PCR and quantitative RT-PCR analysis of *IRS1* and *IRS2* expression in SMMC-7721 cells treated with or without AFB1. AFB1 did not induce changes in either *IRS1* or *IRS2* transcription (Figure 2A). To examine the possibility that AFB1 might affect the turnover rate of IRS1 and IRS2 protein, HepG2 and SMMC-7721 cells were treated with cycloheximide to inhibit new protein synthesis for the times indicated, and extracts assessed by western blotting for IRS1, IRS2 and β-actin to control for loading. The levels of IRS1 in AFB1-treated HepG2 cells declined significantly one day after CHX treatment, while IRS1 levels in untreated cells did not decline until 2 days after CHX treatment. In contrast, the levels of IRS2 in untreated HepG2 cells declined significantly one day after CHX treatment, while IRS2 levels in AFB1-treated cells did not decline even 3 days after CHX treatment (Figure 2B). The levels of IRS1 in AFB1-treated SMMC-7721 cells declined significantly one day after CHX treatment, while IRS1 levels in untreated cells did not decline until 3 days after CHX treatment. In contrast, the levels of IRS2 in untreated SMMC-7721 cells declined significantly 2 days after CHX treatment, while IRS2 levels in AFB1-treated cells did not decline even 3 days after CHX treatment (Figure 2B). Similar finding was detected in Chang liver cells (Figure S1A). These data indicated that AFB1 might decline IRS1 stability but increase IRS2 stability. To determine whether AFB1 had effects on IRS1 and IRS2 degradation, we detected the levels of IRS1 and IRS2 in HepG2, SMMC-7721, and Chang liver cells treated with AFB1 and/or proteasome inhibitor MG132. Treatment with MG132 significantly increased IRS2 levels but not IRS1 levels in HepG2 and SMMC-7721 cells treated without AFB1, indicating that IRS2 might undergo proteasomal degradation in AFB1-untreated hepatoma cells. Treatment with MG132 abrogated the downregulation of IRS1 by AFB1 (Figure 2C and Figure S1B). While both AFB1 and MG132 induced IRS2 accumulation, combination of AFB1 and MG132 did not further increase the levels of IRS2 (Figure 2B and Figure S1B). These data indicate that AFB1 might inhibit IRS2 degradation but promote IRS1 degradation.

### AFB1 induces Akt and Erk1/2 phosphorylation in IGF-IR and IRS2-dependent manner

**Figure 4:**
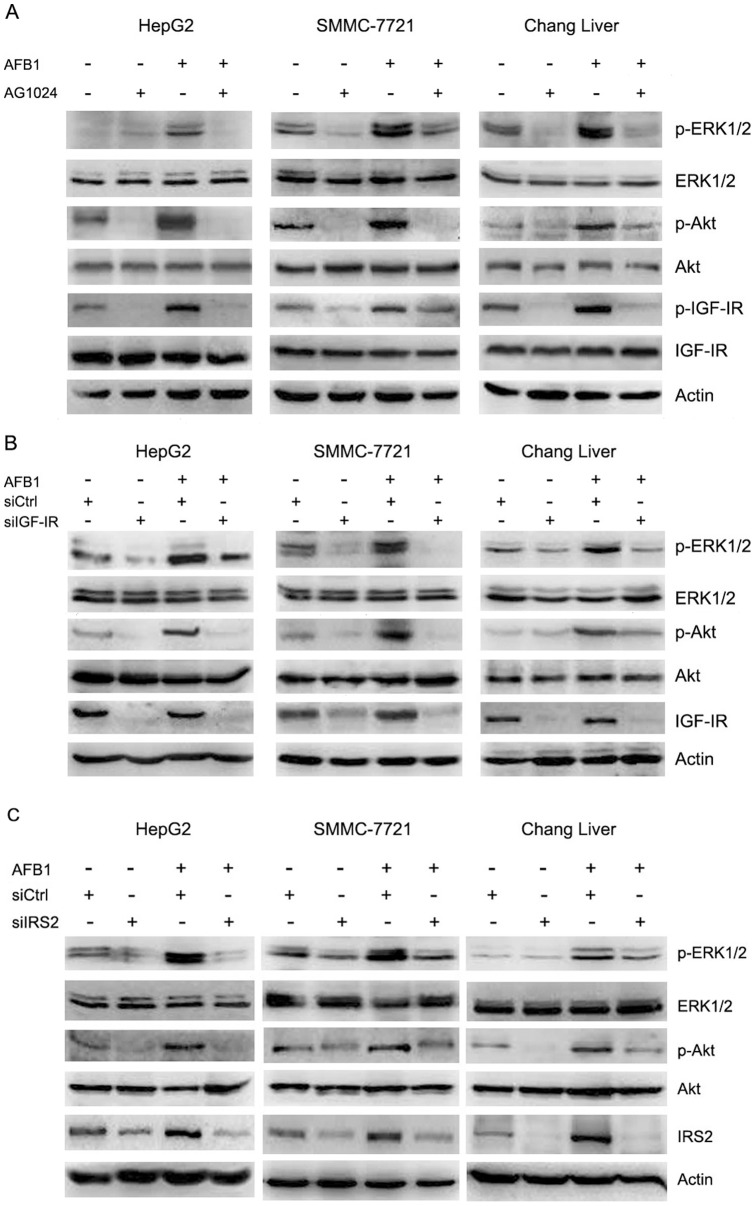
Inhibition of IGF-IR and IRS2 suppresses AFB1-induced Akt and Erk1/2 phosphorylation. (**A**) HepG2, SMMC-7721, and Chang liver cells were treated with or without 2.5 µM AFB1 and 10 µM IGF-IR inhibitor AG1024 for 3 days, followed by western blot analysis of Akt and phosphorylated Akt, Erk1/2 and phosphorylated Erk1/2, IGF-IR and phosphorylated IGF-IR. (**B**) HepG2, SMMC-7721, and Chang liver cells were transfected with control siRNA (siCtrl) or IGF-IR siRNA (siIGFIR). Twenty-four hours later, the cells were treated with or without 2.5 µM AFB1 for 3 days. Cell lysates were subjected to western blot analysis of Akt and phosphorylated Akt, Erk1/2 and phosphorylated Erk1/2, IGF-IR and phosphorylated IGF-IR. (**C**) HepG2, SMMC-7721, and Chang liver cells were transfected with control siRNA (siCtrl) or IRS2 siRNA (siIRS2). Twenty-four hours later, the cells were treated with or without 2.5 µM AFB1 for 3 days. Cell lysates were subjected to western blot analysis of IRS2, Akt and phosphorylated Akt, Erk1/2 and phosphorylated Erk1/2.

Upon IGF-IR phosphorylation and activation, the signals are transduced to PI3K/Akt and MAPK pathways via two adaptor proteins, IRS1 and IRS2. Since AFB1 induced down-regulation of IRS1 and up-regulation of both IGF-IR phosphorylation and IRS2, we investigated the effects of AFB1 on Akt and Erk1/2 phosphorylation. Treatment of HepG2 cells with AFB1 led to an increase in Akt and Erk1/2 phosphorylation (Figure 3A). Similar results were detected in SMMC-7721 and Chang liver cells (Figure 3B and 3C). To determine whether IGF-IR was involved in AFB1-induced Akt and Erk1/2 phosphorylation, we treated HepG2, SMMC-7721 and Chang liver cells with AFB1 and/or AG1024, an inhibitor of IGF-IR and insulin receptor, followed by immunoblot analysis of Akt and Erk1/2 phosphorylation. Treatment with AG1024 suppressed AFB1-induced Akt and Erk1/2 phosphorylation in HepG2, SMMC-7721 and Chang liver cells (Figure 4A and Figure S2A). In addition, we confirmed this finding by transfecting the cells with IGF-IR siRNA . IGF-IR knockdown abrogated AFB1-induced Akt and Erk1/2 phosphorylation (Figure 4B and Figure S2B). These data demonstrated that IGF-IR phosphorylation was responsible for AFB1-induced Akt and Erk1/2 phosphorylation.

Next, we investigated whether up-regulation of IRS2 by AFB1 contributed to Akt and Erk1/2 phosphorylation. Cells were transfected with IRS2 siRNA or control siRNA, followed by treatment with or without AFB1. Immunoblot analysis demonstrated that IRS2 knockdown repressed AFB1-induced Akt and Erk1/2 phosphorylation (Figure 4C and Figure S2C), indicating that up-regulation of IRS2 also contributed to AFB1-induced Akt and Erk1/2 phosphorylation.

### AFB1 stimulates hepatoma cells growth in a dose-dependent manner

**Figure 5:**
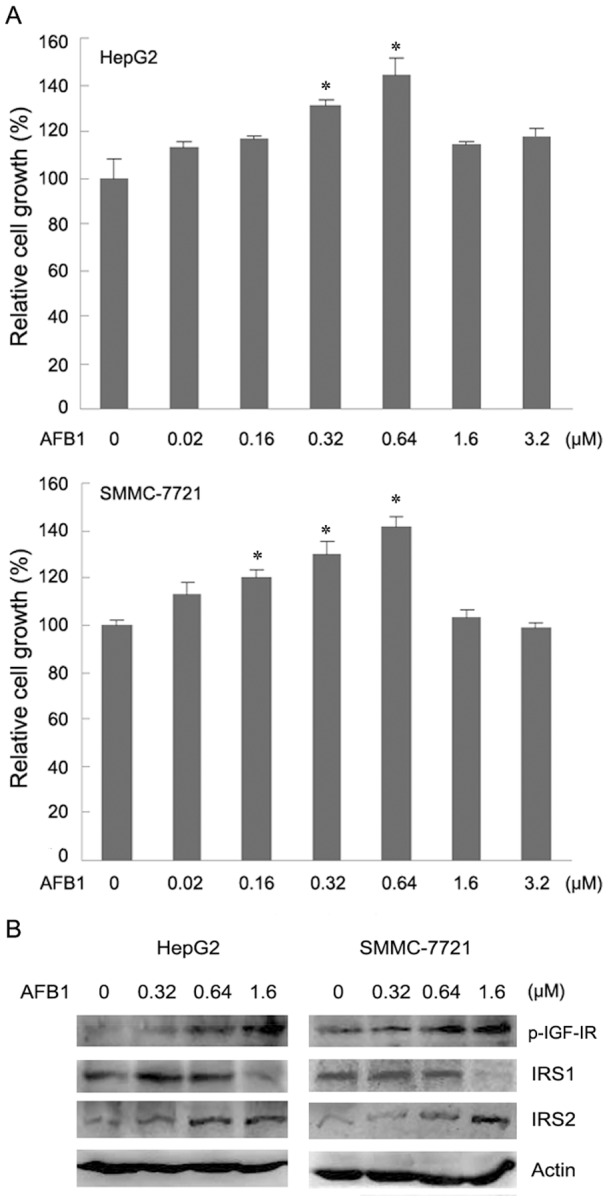
AFB1 stimulates hepatoma cell growth and down-regulates IRS1 in a dose-dependent manner. (**A**) HepG2 and SMMC-7721 cells were plated into 96-well plates, and treated with or without AFB1 at indicated dose for 5 days. Cell growth was detected by CCK-8 reagent. The relative cell growth was plotted. *Bars*, SE. *, *p*<0.05, compared with vehicle-treated cells. (**B**) HepG2 and SMMC-7721 cells were treated with or without AFB1 at indicated dose for 5 days. Cell lysates were subjected to western blot analysis of IRS1, IRS2 and phosphorylated IGF-IR.

IGF1 signaling positively regulates cellular proliferation through both IRS1 and IRS2. Since AFB1 induces IGF-IR phosphorylation, up-regulates IRS2, but down-regulates IRS1, it is unclear how AFB1 may affect hepatoma cell proliferation. We then investigated the effects of AFB1 on hepatoma cells growth. Treatment of HepG2 cells with AFB1 stimulated cell growth in a dose-dependent manner. Treatment of HepG2 cells with 0.32 µM and 0.64 µM of AFB1 moderately stimulated cell growth, but further increase in the dose of AFB1 to 1.6 µM and 3.2 µM resulted in less stimulatory effects on cell growth (Figure 5). Similar findings were observed in SMMC-7721 cells. Whereas treatment of SMMC-7721 cells with 0.16–0.64 µM of AFB1 significantly stimulated cell growth, further increase in the dose of AFB1 to 1.6 µM and 3.2 µM did not stimulate cell growth (Figure 5). To determine whether the decrease in cell growth at higher doses was due to an increase in cell death with increasing doses of AFB1, we detected cell death and apoptosis by trypan blue staining and Hoechst 33342 staining, respectively. No significant difference in cell death or apoptosis was detected in cells treated with above-mentioned dose of AFB1 (data not shown). Previous studies demonstrated that IRS1 was critical for IGF1 induced cell growth. Our data demonstrated that IRS1 was down-regulated by AFB1 at doses more than 1 µM. We then detected the effect of AFB1 at the doses ranging from 0–1.6 µM on IRS1 expression. Treatment of HepG2 and SMMC-7721 cells with 0.32 µM and 0.64 µM of AFB1 did not decrease IRS1 levels. Treatment of HepG2 and SMMC-7721 cells with 1.6 µM of AFB1 led to a decrease in IRS1 expression (Figure 5B). The levels of both IRS2 and phosphorylated IGF-IR tended to increase with elevated doses of AFB1 (Figure 5B). These data demonstrate that the effect of AFB1 on hepatoma cell growth is dose-dependent.

### AFB1 promotes hepatoma cells migration through IGF-IR/IRS2 axis

**Figure 6:**
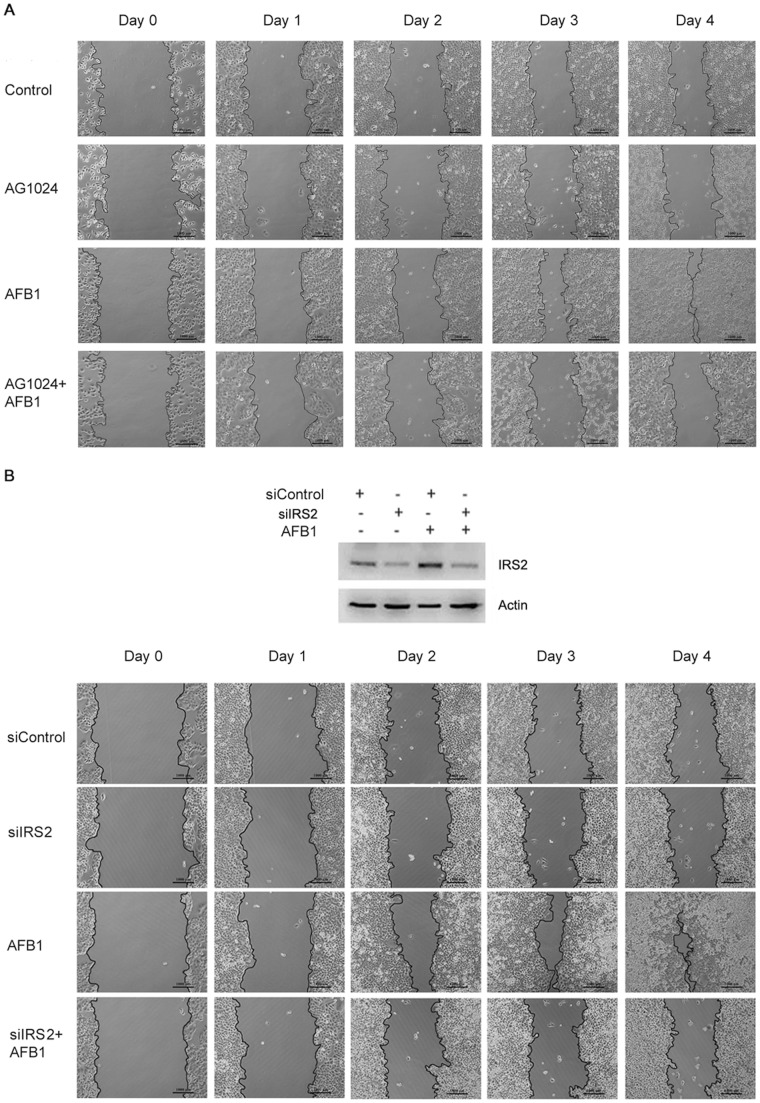
AFB1 stimulates hepatoma cell migration through IGF-IR/IRS2 axis. (**A**) SMMC-7721 cells were seeded into 6-well plates. Upon confluency, scratches were made in cell cultures. To inhibit cell proliferation, the cells were treated with 2 µg/ml mitomycin C. Also, the cells were treated with or without 2.5 µM AFB1 and 10 µM IGF-IR inhibitor AG1024 for 4 days. *Bar*, 1000 µm. (**B**) SMMC-7721 cells were transfected with siCtrl or siIRS2. Twenty-four hours later scratches were made in cell cultures. The cells were treated with 2 µg/ml mitomycin C, and treated with or without 2.5 µM AFB1 for 4 days. *Bar*, 1000 µm. Cell lysates from siCtrl- or siIRS2-transfected cells were harvested and subjected to Western blot analysis of IRS2 expression.

Given that IRS2 is tightly involved in IGF1-induced cell migration, we then detected whether AFB1 could promote hepatoma cell migration through IGF-IR/IRS2 axis. SMMC-7721 cells treated with or without AFB1 and/or AG1024 were subjected to wound-healing assay. Indeed, AFB1 significantly stimulated HepG2 cell migration. Treatment with AG1024 repressed AFB1-induced cell migration (Figure 6A), indicating that activation of IGF-IR was responsible for AFB1-induced cell migration.

To determine roles of IRS2 in AFB1-induced cell migration, SMMC-7721 cells were transfected with IRS2 siRNA or control siRNA, followed by treatment with AFB1. Wound-healing assay demonstrated that IRS2 knockdown abrogated AFB1-induced cell migration (Figure 6B). These data strongly demonstrate that IRS2 has key roles in AFB1-induced hepatoma cell migration.

## Discussion

Previous studies have demonstrated that AFB1 is a potent carcinogen that could induce mutagenesis. After being metabolized in liver cells, activated AFB1 induces base substitution mutations in DNA [14]. The most prominent mutations in AFB1-treated mice are G:C to T:A transversions and G:C to A:T transition [15]. Notably, a G to T mutation at the 3^rd^ position of codon 249 of the *p53* tumor suppressor gene has been observed in over 50% of patients with HCC associated with high exposure to AFB1 [16]. Although codon 249 is the best-known location where *p53* is mutated in HCC cases with AFB1 exposure, a study in human fibroblasts show that AFB1 can induce *p53* mutations at codons other than codon 249 [17]. Given that *p53* plays key roles in suppressing tumorigenesis, it is conceivable that *p53* mutation is important mechanism underlying AFB1-induced carcinogenesis [9]. However, the mechanisms underlying AFB1 hepatocarcinogenesis may not be limited to *p53* mutations. A recent study reportedly showed that AFB1 upregulated both IGF2 and IGF-IR expression in hepatoma cells thereby activating IGF-IR signaling pathway [8]. Deregulation of IGF signaling is linked to tumorigenesis. IGF-IR is frequently over-expressed or activated in a variety of human tumors. In our current study, we did find that AFB1 induced IGF-IR phosphorylation. However, we did not find that AFB1 up-regulated IGF1, IGF2 (data not shown) and IGF-IR expression, which was not in agreement with Ubagai's study. The mechanisms underlying AFB1 induction of IGF-IR phosphorylation remains to be studied. The direct activation of IGF-IR by its ligands such as IGF1 and IGF2 is fast, usually after several minutes. However, the phosphorylation of IGF-IR by AFB1 was not a short time effect (data not shown), and actually detected after several days, indicating that the activation of IGF-IR by AFB1 might be indirect. Moreover, our current study demonstrated that AFB1 reciprocally regulated IRS1 and IRS2 levels. Up-regulation of IRS2 contributed to AFB1 induced activation of Akt and ERK1/2 thereby stimulating hepatoma cell proliferation and migration.

Mounting studies have reported that elevated IGF or insulin in the serum correlated with increased risk of cancer development [11]. IGF-IR signaling is frequently up-regulated in a variety of human tumors. Activation of IGF-IR signaling may promote tumor progression by stimulating cell growth, migration and invasion. IRS1 and IRS2 are cytoplasmic adaptor proteins that functions downstream of activated IGF-IR. Although IRS1 and IRS2 share considerable homology, they have non-redundant functions in development and metabolism [18], [19]. Both IRS1 and IRS2 stimulate cellular proliferation. Overexpression of either IRS1 or IRS2 can promote mammary tumorigenesis. However, IRS1 and IRS2 may have divergent functions in cancer metastasis. IRS1 deficient tumor cells are more invasive, while IRS2 deficient tumor cells are less invasive [20], [21]. IGF1 promotes cell motility in human breast carcinoma cell lines that predominately express IRS2 [22]. In addition, IRS2 promotes cell motility and invasion in neuroblastoma and mesothelioma cells, while overexpres­sion of IRS1 in prostate carcinoma cells decreases tumor cell motility [23], [24]. These data suggest that a high IRS2/IRS1 ratio may correlate with tumor aggressiveness. Previous studies showed that IRS1 was down-regulated in non-small cell lung tumors and poorly differentiated, ER(-) breast carcinoma [25], [26]. IRS2 expression is up-regulated in hepatocellular carcinoma [27]. Our current study demonstrates that AFB1 up-regulates IRS2 but down-regulates IRS1 levels in hepatoma cells, leading to a shift in IRS1 and IRS2 expression towards a high IRS2/IRS1 ratio. Consistent with studies in other models, our current study show that AFB1 stimulates hepatoma cell migration in IGF-IR- and IRS2-dependent manner. IRS1 loss and IRS2 accumulation may represent important mechanism underlying AFB1-induced tumor progression. Accumulated IRS2 may compensate for the loss of IRS1 and enhance hepatoma cell migration by modulating cell migration-promoting effectors that are shared or not shared by IRS1. However, our data show that AFB1 stimulates cell proliferation at relatively lower dosages and does not stimulate cell growth at higher dosages that significantly up-regulate IRS2 but down-regulate IRS1, indicating that IRS1 may be essential for stimulating cellular proliferation and that IRS2 may be unable to compensate for the loss of IRS1 to stimulate cell proliferation, even though IGF-IR, Akt and Erk are activated. Therefore, AFB1 may not only induce the transformation of liver cells, but also stimulate the proliferation and migration of cancer cells thereby accelerating tumor progression. AFB1 often synergizes with other etiological factors such as HBV infection to induce hepatocarcinogenesis. We speculate that AFB1 may promote existing tumor foci by stimulating IGF-IR signaling, no matter what induces these tumor foci.

Since AFB1 has potent carcinogenecity and genotoxicity, chemoprevention of AFB1 hepatocarcinogenesis has been an attractive topic. A major strategy to prevent AFB1 hepatocarcinogenesis is to target AFB1 metabolism. The AFB1 aldehyde reductase and the aflatoxin-conjugating class-α glutathione S-transferase (GST) A5 subunit can be induced by phytochemicals such as benzyl isothiocyanate, coumarin, or indole-3-carbinol, and drugs such as oltipraz and phenobarbital. Induction of hepatic aldo-keto reductase activity toward AFB1-dialdehyde and GST activity toward AFB1-8,9-epoxide can help to detoxify AFB1 metabolites and inhibit carciogenesis. Dietary intervention with coumarin reportedly inhibits the number and size of tumors induced by AFB1 in animal models [28]. Given that our study demonstrates that AFB1 also upregulates IGF-IR signaling and induces IRS2 accumulation, this pathway may also be a feasible target to suppress AFB1 carcinogenesis. Combination of chemicals that interfere with AFB1 metabolism and drugs that inhibit IGF-1 signaling may synergistically suppress AFB1-induced carcinogenesis.

## Materials and Methods

### Reagents and antibodies

AFB1 was purchased from Sigma-Aldrich Inc. (St. Louis, MO, USA) and prepared by reconstituting in DMSO and by diluting to the appropriate concentration and stored at −20°C. Tyrphostin AG1024 was purchased from Sigma-Aldrich Inc. (St. Louis, MO, USA). The PI3K inhibitor LY294002, anti-IGF-IRβ (#3027), anti-phosphorylated IGF-IRβ (#3024; Tyr 1135/1136), anti-Akt (#3063) and phosphorylated Akt (#4060; Ser 473)135/1136)nclear whether doses more than 1, anti-IRS1 (#2382), anti-Erk1/2 (#4695) and phosphorylated Erk1/2 (#9101; Thr 202/204) antibodies were purchased from Cell Signaling Technology (Beverly, MA, USA). Anti-actin antibody was from Santa Cruz Biotechnology (#sc-1616; Santa Cruz, CA, USA).

### Cell culture

Hepatoma cell lines HepG2 and SMMC-7721, and the immortalized human liver cell line Chang liver were obtained from Cell Lines Bank, Chinese Academy of Science (Shanghai, China). The cell lines have been tested and authenticated by DNA (STR) profiling. The cells were maintained in Dulbecco minimal essential medium (DMEM) containing 10% fetal bovine serum and 50 units/ml of penicillin and 50 µg/ml streptomycin sulfate, and incubated at 37°C in a humidified atmosphere of 5% CO_2_.

### Transfection of siRNA

All siRNA are custom-synthesized products of Ribobio Co., Ltd. (Guangzhou, China). The target sequences used for knockdown of IGF-IR and IRS2 was 5′-CAATGAGTACAACTACCGC-3′ and 5′-GTACATCAACATCGACTTT-3′, respectively. The double-stranded siRNA duplex was dissolved in DEPC-treated water. For transfection, cells were plated into culture dishes and incubated for two days. LipofecTAMINE 2000 reagent (Invitrogen, Carlsbad, CA, USA) was diluted in Opti-MEM I Reduced Serum Medium and incubated at room temperature for 5 min. In addition, siRNA duplex was diluted in Opti-MEM I Reduced Serum Medium and mixed with the pre-diluted LipofecTAMINE 2000. The mixture was incubated at room temperature for 20 min and then split into culture dishes with a final concentration of 50 nmol/L of siRNA.

### Reverse transcription-polymerase chain reaction (RT-PCR) and quantitative real-time RT-PCR

Total RNA was extracted using TRIzol reagent (Invitrogen). First strand cDNAs were synthesized using the Moloney murine leukemia virus reverse transcriptase and oligo(dT) primers. The primer sequences for human *IRS1* were 5′- TGCACTGTGACACCAGAATAAT-3′ (forward) and 5′- CGCCCACATTGTTCATTCCAA -3′ (reverse). The primer sequences for human *IRS2* were 5′-GCTGCTGCTACAGCTCCT-3′ (forward) and 5′-GGCTCGCCAAAGTCGATGT-3′ (reverse). *All targets* were amplified by PCR using PCR amplification mix and 200 nM primers. The PCR conditions for both sets of primers were as follows: first cycle at 94°C for 2 min, then 30 cycles of 94°C for 30 s, 52°C for 30 s, and 72°C for 30 s and a final elongation at 72°C for 7 min.

For real-time RT-PCR, *IRS1* and *IRS2* were amplified by real-time PCR using the SYBRGreen PCR amplification mix (total volume 25 µl) and 200 nM primers. *GAPDH* was also amplified as a reference gene. Relative quantification with the comparative threshold cycle(Ct) was done using the Ct method. The amount of *IRS1* or *IRS2* gene normalized to the endogenous reference gene (*GAPDH*) is given by 2^−ΔCt^, where ΔCt is Ct (*IRS1* or *IRS2*) – Ct(*GAPDH*). The PCR conditions for both sets of primers were as follows: first cycle at 95°C for 4 min, then 35 cycles of 95°C for 30 s, 57°C for 30 s, and 72°C for 30 s and a final elongation at 72°C for 10 min.

### Western blotting

Cells were lysed in cold lysis buffer (50 mM Tris pH 7.4, 150 mM NaCl, 1% NP-40, 1% Triton X-100, 0.1% SDS, 1% sodium deoxycholate, 1 mM EDTA, 50 mM NaF, 10 mM sodium pyrophosphate, 0.5 mM DTT) with freshly added protease inhibitors (Sigma Chemical Company, St Louis, MO, USA). Aliquots of proteins were boiled in 2× loading buffer for 10 min, then loaded into 10% Tris-HCl-Polyacrylamide gels, and transferred to PVDF membrane (Millipore Corporation, Billerica, MA, USA). Membranes were incubated with primary antibodies and appropriate HRP-secondary antibodies. Detection was performed with chemiluminescent agents. The immunoblots were subjected to densitometric quantification.

To examine IRS1 and IRS2 decay in hepatoma cells, the cells were treated with 25 µg/ml cycloheximide (CHX) for 1–3 days to inhibit new protein synthesis, and IRS1, IRS2, β-actin levels assessed by western blotting. The immunoblots were subjected to densitometric quantification.

### Detection of cell growth

Hepatoma cells were plated in 96-well plates at 1,500 cells per well. The next day, cells were treated with or without AFB1 in four replicates. After 5 days, viable cells were assessed by incubating cells with Cell Counting Kit-8 (CCK-8) reagents (Dojindo Laboratory Co., Ltd., Kumamoto, Japan) for 2–4 h and measuring the absorbance at 490 nm , and at 630 nm as reference, with a microplate reader (Bio-Rad, Hercules, CA, USA).

### Wound-healing assay

Cells were seeded in 6-well plates at 50,000 cells/well. Twenty-four hours after the plating, the cells were treated with 2 μg/mL mitomycin C to inhibit cell proliferation. The IRS2 siRNA (siIRS2) or control siRNA (siCtrl) was transfected into the cells. Twenty-four hours after transfection, a 1-mm scrape was placed through the middle of the confluent cultures with a pipette tip and washed twice with PBS to remove debris, followed by treating with or without 2.5 μmol/L AFB1. The wound was observed under a phase-contrast microscope everyday.

### Statistical analysis

One-way ANOVA with least significant difference post hoc test (SPSS 13.0 for Windows) was used to test for the differences. All statistical tests were two-tailed, and difference to be considered statistically significant when *p*<0.05.

## Supporting Information

Figure S1 **AFB1 affects IRS1 and IRS2 turnover in Chang liver cells.** (**A**) Chang liver cells were treated with 25 µg/ml CHX to inhibit new protein synthesis for the times indicated. In parallel, the cells were treated with combination of AFB1 and CHX. Total proteins were harvested and subjected to western blotting for IRS1, IRS2 and β-actin to control for loading. (**B**) Chang liver cells were treated with or without 2.5 µM AFB1 and 2 µM proteasome inhibitor MG132 for 3 days, followed by western blot analysis of IRS1 and IRS2 levels.
(TIF)

Figure S2 **Densitometric analysis of the effects of Inhibition of IGF-IR and IRS2 on AFB1-induced Akt and Erk1/2 phosphorylation.** (**A**) HepG2, SMMC-7721, and Chang liver cells were treated with or without 2.5 µM AFB1 and 10 µM IGF-IR inhibitor AG1024 for 3 days, followed by western blot analysis of Akt and phosphorylated Akt, Erk1/2 and phosphorylated Erk1/2, IGF-IR and phosphorylated IGF-IR. Immunoblots were subjected to densitometric analysis. The relative levels of Akt, phosphorylated Akt, Erk1/2, phosphorylated Erk1/2 and phosphorylated IGF-IR after normalization to actin were plotted. The relative levels of target proteins in cells treated without AFB1 and AG1024 were set as 1. A statistical analysis of densitometric quantification of immunoblots from individual experiments was shown. *, *p*<0.05. (**B**) HepG2, SMMC-7721, and Chang liver cells were transfected with control siRNA (siCtrl) or IGF-IR siRNA (siIGFIR). Twenty-four hours later, the cells were treated with or without 2.5 µM AFB1 for 3 days. Cell lysates were subjected to western blot analysis of Akt and phosphorylated Akt, Erk1/2 and phosphorylated Erk1/2, IGF-IR and phosphorylated IGF-IR. Immunoblots were subjected to densitometric analysis. The relative levels of Akt, phosphorylated Akt, Erk1/2, phosphorylated Erk1/2, and IGF-IR after normalization to actin were plotted. The relative levels of target proteins in cells treated with siControl were set as 1. A statistical analysis of densitometric quantification of immunoblots from individual experiments was shown. *, *p*<0.05. (**C**) HepG2, SMMC-7721, and Chang liver cells were transfected with control siRNA (siCtrl) or IRS2 siRNA (siIRS2). Twenty-four hours later, the cells were treated with or without 2.5 µM AFB1 for 3 days. Cell lysates were subjected to western blot analysis of IRS2, Akt and phosphorylated Akt, Erk1/2 and phosphorylated Erk1/2. Immunoblots were subjected to densitometric analysis. The relative levels of Akt, phosphorylated Akt, Erk1/2, phosphorylated Erk1/2, and IRS2 after normalization to actin were plotted. The relative levels of target proteins in cells treated with siControl were set as 1. A statistical analysis of densitometric quantification of immunoblots from individual experiments was shown. *, *p*<0.05.
(TIF)
