# Supplementary figures and images for: Aflatoxin B1 Up-Regulates Insulin Receptor Substrate 2 and Stimulates Hepatoma Cell Migration
Source: PLoS One. 2012 Oct 24;7(10):e47961. doi: 10.1371/journal.pone.0047961 (PMC3480444; doi:10.1371/journal.pone.0047961)

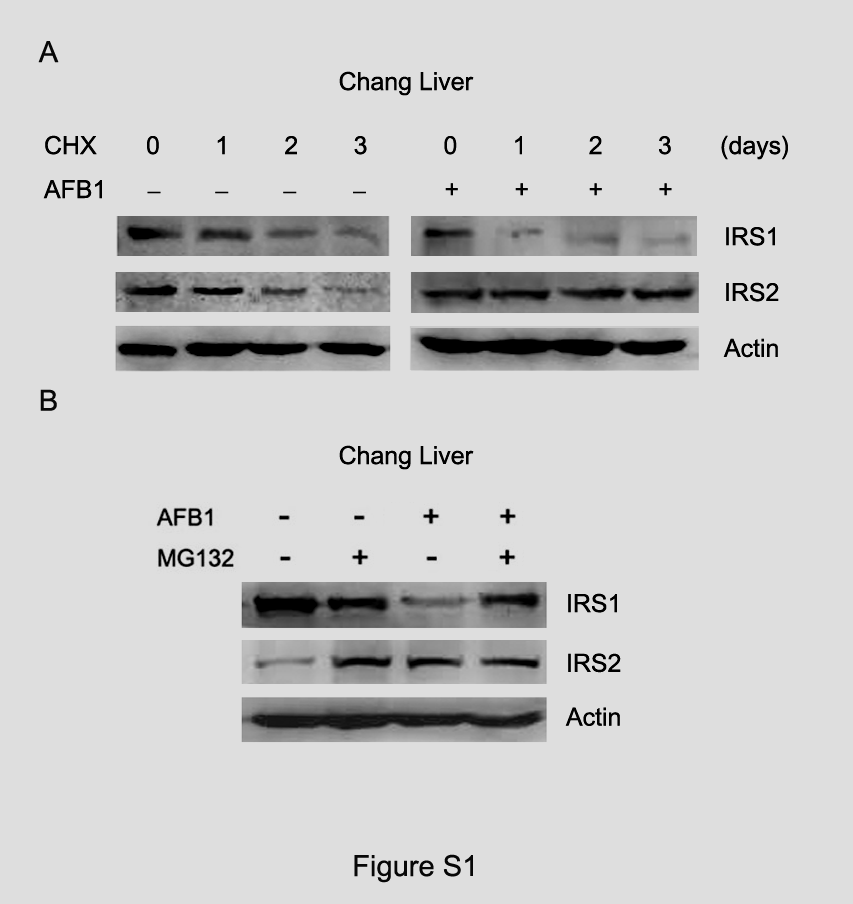

Supplement: Figure S1 — AFB1 affects IRS1 and IRS2 turnover in Chang liver cells. (A) Chang liver cells were treated with 25 µg/ml CHX to inhibit new protein synthesis for the times indicated. In parallel, the cells were treated with combination of AFB1 and CHX. Total proteins were harvested and subjected to western blotting for IRS1, IRS2 and β-actin to control for loading. (B) Chang liver cells were treated with or without 2.5 µM AFB1 and 2 µM proteasome inhibitor MG132 for 3 days, followed by western blot analysis of IRS1 and IRS2 levels. (TIF) [file pone.0047961.s001.tif]

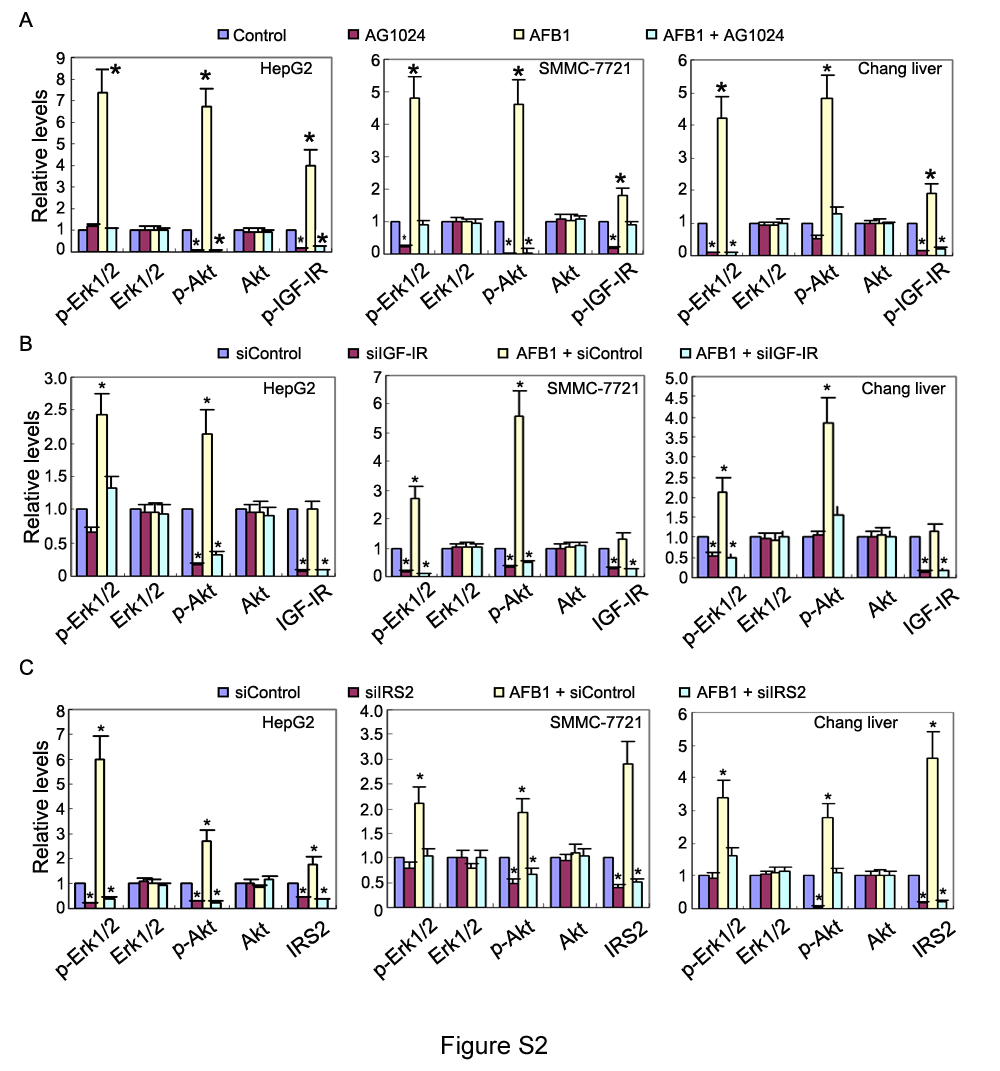

Supplement: Figure S2 — Densitometric analysis of the effects of Inhibition of IGF-IR and IRS2 on AFB1-induced Akt and Erk1/2 phosphorylation. (A) HepG2, SMMC-7721, and Chang liver cells were treated with or without 2.5 µM AFB1 and 10 µM IGF-IR inhibitor AG1024 for 3 days, followed by western blot analysis of Akt and phosphorylated Akt, Erk1/2 and phosphorylated Erk1/2, IGF-IR and phosphorylated IGF-IR. Immunoblots were subjected to densitometric analysis. The relative levels of Akt, phosphorylated Akt, Erk1/2, phosphorylated Erk1/2 and phosphorylated IGF-IR after normalization to actin were plotted. The relative levels of target proteins in cells treated without AFB1 and AG1024 were set as 1. A statistical analysis of densitometric quantification of immunoblots from individual experiments was shown. *, p<0.05. (B) HepG2, SMMC-7721, and Chang liver cells were transfected with control siRNA (siCtrl) or IGF-IR siRNA (siIGFIR). Twenty-four hours later, the cells were treated with or without 2.5 µM AFB1 for 3 days. Cell lysates were subjected to western blot analysis of Akt and phosphorylated Akt, Erk1/2 and phosphorylated Erk1/2, IGF-IR and phosphorylated IGF-IR. Immunoblots were subjected to densitometric analysis. The relative levels of Akt, phosphorylated Akt, Erk1/2, phosphorylated Erk1/2, and IGF-IR after normalization to actin were plotted. The relative levels of target proteins in cells treated with siControl were set as 1. A statistical analysis of densitometric quantification of immunoblots from individual experiments was shown. *, p<0.05. (C) HepG2, SMMC-7721, and Chang liver cells were transfected with control siRNA (siCtrl) or IRS2 siRNA (siIRS2). Twenty-four hours later, the cells were treated with or without 2.5 µM AFB1 for 3 days. Cell lysates were subjected to western blot analysis of IRS2, Akt and phosphorylated Akt, Erk1/2 and phosphorylated Erk1/2. Immunoblots were subjected to densitometric analysis. The relative levels of Akt, phosphorylated Akt, Erk1/2, phosphorylated Er [file pone.0047961.s002.tif]
